# Health of the Pig1Read before the Illinois Swine-breeders’ Association.

**Published:** 1896-03

**Authors:** D. McIntosh

**Affiliations:** Urbana, Ill.


### HEALTH OF THE PIG.[1]

BY D. McINTOSH, V.S.,
URBANA, ILL.

EDMOND PARK says: "If we had a perfect knowledge of the laws of life and could apply this knowledge in a perfect system of hygienic rules, disease would be impossible. Hygiene is the art of preserving health. It aims at rendering growth more perfect, decay less rapid, life more vigorous, death more remote." So beautiful and comprehensive is this definition it ought to be often repeated.

In dealing with this subject of health there are several things to be taken into consideration; this I will do as briefly as possible. First, we should follow nature's steps as closely as practicable, and should consider the condition of the pig in its natural haunts and deprive it of as few of them as possible. The pig is an omnivorous animal and eats all. It is destined by nature to uproot plants and grope among the dropped acorns and other fruits of the forest; and Youatt says: "In point of fact the snout of the pig is its spade with which it roots in the ground for roots and earth worms."

By putting an iron ring through the cartilage of its nose we thus deprive it of the power of searching for and analyzing its food, and by doing so we prevent it from getting substances which would be very beneficial for the maintenance of its health. To be profitable it is necessary to feed pigs more food than they could obtain in a natural state in order to bring them to maturity as fast as possible, and this is done at the expense of the animal's health; seeing that this has to be done we ought to consider what kind of food is best to obtain this result and at

[1] Read before the Illinois Swine-breeders' Association.

the same time keep the animal in a vigorous condition. Yeo says that if an animal is in perfect health the pure alkaline blood circulating through the tissues of the body prevents the germs of disease from finding a suitable place to develop. Let us look for a short time at the physiological actions of some of the most important organs of the animal body, as we will then be better able to understand some of the causes of ill health.

The stomach of the pig in its natural state is small and the intestines have great assimilating power. In this capacity the pig is ahead of all other animals, which accounts for its taking on fat so rapidly. By giving large quantities of food the stomach becomes distended and in some cases weakened so that it cannot digest the food properly, and it passes out of the stomach in this condition into the intestines, where it acts as a foreign body, setting up disturbance, deranging the mucous membrane, leaving it in a condition favorable for the development of microbes and other germs of disease; the undigested portion will pass out as feces. The pig should be fed as much during the fattening period as it can digest, and nothing more. This can be easily ascertained by examining the feces. The kidneys secrete the urine and other effete material, the result of the disintegration of the nitrogenous substances in the body; they require to be in a healthy, active state to perform this function, or blood-poisoning is the result; if not blood-poisoning, sufficient disturbance is caused to leave the animal liable to disease. The heart should be strong and vigorous in order to be able to propel the blood to all the tissues of the body to nourish them. The lungs should be strong, with large capacity to draw in oxygen and give off carbon dioxide and other effete materials, in this way keeping the blood pure. The nerves which govern all parts of the body should also be strong and active. This is largely accomplished by the kind of food we feed the animal on.

What is the animal body composed of? The chemical constituents of the animal body may be thus classified: First, albuminous substances characterized by the presence of nitrogen, carbon, hydrogen, and oxygen. Second, carbohydrates and hydrocarbons, characterized by the absence of nitrogen and the presence of carbon, hydrogen, and oxygen. Third, salts and water. In order to keep all the tissues of the body in healthy action and vigor it is necessary to see that the animal gets a food which contains all these elements, or to give a mixed diet which will combine to furnish the materials necessary. Food should be composed of nitrogenous portions called albuminates or flesh-makers, hydrocarbons or fat-makers, carbohydrates, which are starch and sugar bodies, also fat-producers. These are all necessary for the healthy development of the animal tissues. Let us see which of the various grains contain the substances mentioned.

| | Corn. | Oats. | Peas. | Red Clover. |
|---|---|---|---|---|
| Water | 13·9 | 13·5 | 13·3 | 16·7 |
| Albumin | 10·1 | 11·9 | 22·4 | 13·4 |
| Fats | 4·8 | 5 8 | 2·5 | 3 2 |
| Carbohydrates and non-nitrogenous extractive matters | 66·8 | 57·5 | 52·3 | 29·9 |
| Cellulose | 2·8 | 8·1 | 9·2 | 35·8 |
| Ash | 1·7 | 2·6 | 2·5 | 2 6 |

These figures vary considerably according to the condition of the ground on which the grains grow, whether it is rich or poor, cultivation, etc. The above table shows that oats and peas are more evenly balanced than corn. They are therefore the grains best suited for the growth and development of the tissues of the body, and also to keep them in a healthy state. When food substances are deficient in the albuminates and salts the system is generally lowered in tone, and there is a tendency to the formation of "exudations," composed of imperfectly developed cells, which in the great majority of cases, from the very beginning, are incapable of development into perfect entities, having only one potential quality, that of dying, and in so doing cause various derangements in the body, especially in the respiratory organs, producing tuberculosis and affections of the glands of the intestines. Oats also contain a nitrogenous alkaloid called avenin, which possesses the property of acting as a nerve stimulant. It is on this account that horses largely fed on oats are so spirited. The salts or ash that these substances contain are all needed in the animal body in order that they will grow, and also support the system in older animals. Oats is the grain par excellence for the horse and peas for the pig. Corn, alone, has not sufficient albuminates and salts and has too much starchy substance, which is converted into fat and is therefore a grain which is not fit food for a young growing animal. It is necessary to feed other materials which contain albuminates to supply the deficiency of this material in the corn. And I am satisfied that the prevalence of cholera among pigs in the corn-growing States is in great part due to the feeding of too much corn. In Canada, where the pig is mostly fed on peas and oats and the refuse of wheat and rye, cholera is unknown. It is true there have been a few cases of cholera in Canada, but it has been mostly on the borders where it was supposed to have been brought over the river, and some years ago at Montreal, supposed to have been caused by feeding on distillery slops. Messrs. Lawes and Gilbert made a number of experiments on feeding in England, and found that pigs fed exclusively on corn would frequently swell in the neck. They did not wish to discontinue the experiment, and therefore resolved to try the effect of putting some mineral substance in a trough within reach of the pigs. They made a mixture of twenty pounds of sifted coal ashes, four pounds of common salt, and one pound of superphosphate of lime. A trough containing this mineral mixture was put into the pen at the commencement of the second fortnight, and the pigs began to lick it with evident relish. From this time the swellings or tumors, as well as the difficulty in breathing, began to diminish rapidly, and at the end of a month had entirely disappeared. The three pigs consumed of the mineral mixture described above nine pounds during the first fortnight, six pounds during the second, and nine pounds during the third. This, although a single experiment, shows, I think, that these animals may be fed on corn with impunity, providing that a compound of this or some other be put within their reach. I would suggest the following:

First, that we should avoid inbreeding as much as possible, as there is no doubt that it lessens the vitality of the offspring, leaving them in a condition liable to disease.

Second, that we select large sows, well developed, and at least one year old.

Third, that the boar should be of a smaller breed, compact, and of a vigorous constitution. This combination will insure strong, healthy offspring.

Fourth, that the sow and the boar should be fed on ground oats and bran mixed, sufficient to keep them growing, but not too fat, as when they are too fat their vitality is lessened. They should have a small field to run in, separate, at some distance from each other. They should not have rings in their noses, but be allowed to dig at pleasure, as they will find material in the ground useful for their health. If they should show signs of getting fat, cut down their feed; on the other hand, if they are losing flesh, feed a little more. They should have a shelter from the sun in summer and a comfortable place to sleep in at night in winter. They should have green clover in summer and dry clover-hay in winter. Give plenty of fresh water and a little salt mixed with their food. Pigs treated in this way will seldom have any ailments.

Fifth, that having strong, healthy, young pigs to begin with, it is necessary to feed them on materials that will keep up vigor and at the same time produce rapid growth. This can be accomplished by feeding them on ground oats or peas mixed with bran, and turning into a clover field, if possible; if not, clover should be cut and brought to them. Milk of all kinds is useful. They should have a field to roam in, and after they are old enough the boars should be separated from the sows. The above food contains all the elements necessary for the growth and development of the pig. The bran, shell of the oats, and the clover contain a large percentage of cellulose, and although the pig cannot digest more than half of this material, yet it is very useful, as it contains just what is needed to assist in forming the tissues of the body. Pigs fed as above will have all parts of their bodies well nourished and in a state of vigor to perform all the functions required of them to fortify their body against at least ordinary diseases.

Sixth, that too many pigs should not be kept together, as they are apt to sleep in the same place, and, although it may be well ventilated or even out in the open air, they are apt to breathe some of the foul air emanating from their bodies. No class of animals thrive well where numbers are kept together. When the time arrives to feed the hogs for market you will have a splendid foundation to begin feeding on: strong digestive and assimilating organs which will be able to digest and assimilate large quantities of food. Corn can now be used, with a little ground oats and bran, with advantage and profit. I think, if this method were carried out, in a few years hog cholera would be a thing of the past. Gentlemen, try it.

